# Supplementary material for: Parallel interrogation of the chalcogenide-based micro-ring sensor array for photoacoustic tomography
Source: Nat Commun. 2023 Jun 5;14:3250. doi: 10.1038/s41467-023-39075-3 (PMC10241812; doi:10.1038/s41467-023-39075-3)
Supplement: Supplementary file 1 — Supplementary Information [file 41467_2023_39075_MOESM1_ESM.pdf]

Supplementary Information for "Parallel interrogation of the chalcogenide-based micro-ring sensor array for photoacoustic tomography"

Jingshun Pan[1,2,3 #], Qiang Li[1, #], Yaoming Feng[1], Ruifeng Zhong[1], Zhihao Fu[1], Shuixian Yang[1], Weiyuan Sun[1], Bin Zhang[1,2], Qi Sui[2], Jun Chen[1], Yuecheng Shen[1,2,*] & Zhaohui Li[1,2,*]

[1]*School of Electronics and Information Technology, Guangdong Provincial Key Laboratory of Optoelectronic Information Processing Chips and Systems, Sun Yat-sen University, Guangzhou 510275, China*

[2]*Southern Marine Science and Engineering Guangdong Laboratory (Zhuhai), Zhuhai 519000, China*

[3]*Guangdong Provincial Key Laboratory of Nanophotonic Functional Materials and Devices, South China Normal University, Guangzhou 510006, China*

[#]These authors contributed equally to this work

*Email: shenyuecheng@mail.sysu.edu.cn; lzhh88@mail.sysu.edu.cn

Supplementary Note 1. Detailed imaging procedures for biological samples

The experimental setup for imaging biological tissue is schematically shown in Supplementary Fig. 1. The light source was chosen as a 532-nm laser (Beamtech, Dawa 100) with a pulse width of 6.5 ns and a repetition rate of 10 Hz. An optical diffuser (Thorlabs, DG10-120) was used to expand and homogenize illuminating light. The light illuminated the sample from the top with an area of about 8 mm in diameter. To mitigate artifacts due to the limited view, the water tank was mounted on a motorized rotational stage, which rotated with a step size of 1 degree. The sensor array was hung in water at the same horizontal plane and was about 4 cm away from the edge of the sample. In this condition, photoacoustic tomography (PAT) was performed by rotating the zebrafish while keeping the sensor array and excitation light fixed. When the pulsed light irradiated the sample, each micro-ring sensor produced an A-line signal, which contains 4,096 data points. After rotating 360 degrees, universal back projection [1] was employed to reconstruct the image through these 360 sets of A-line signals. To acquire one image, the scanning process took about 36 seconds (360 optical pulses). A microcontroller (STMicroelectronics, STM32) was employed to synchronize the motorized rotational stage, the laser, and the data acquisition process.

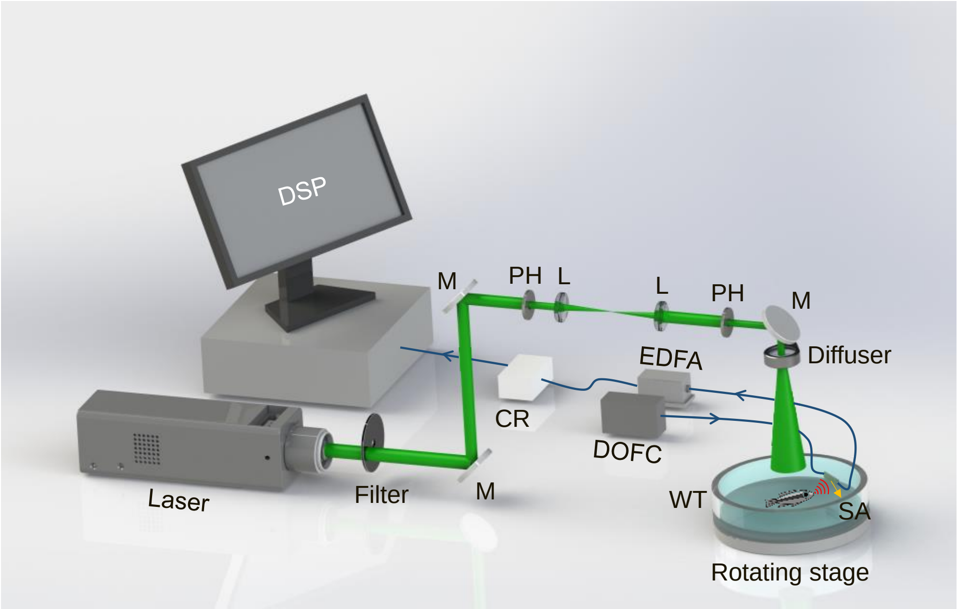

Supplementary Fig. 1. Experimental setup of performing photoacoustic tomography for biological samples. M: mirror; PH: pinhole; L: lens; SA: sensor array; WT: water tank; DOFC: digital optical frequency comb; EDFA: erbium-doped fiber amplifier; CR: coherent receiver; DSP: digital signal processing.

Supplementary Note 2. The protocol to prepare the leaf

This protocol describes the detailed procedure to prepare the leaf for imaging purposes. The main goal of these procedures is to remove mesophyll through chemical corrosion using acidic or alkaline substances. The leaf veins, on the other hand, are kept and stained for imaging purposes. The protocol is illustrated as follows:

1. Preparing solution: add 20 g sodium hydroxide and 10 g sodium carbonate into 500 ml water and stir them evenly.
2. Heating solution: when the solution is about to boil, add a piece of a diamond leaf. While keeping the solution slightly boiling, heat the diamond for 5 minutes.
3. Removing mesophyll: take out the diamond leaf and put it into clear water. Use a brush to gently remove the mesophyll along the directions of the veins. Then, clean the leaf with clean water and dry the water again.
4. Bleaching: put the leaf into sodium hypochlorite solution for bleaching, wash them with clean water after bleaching, and dry the water again.
5. Staining: dilute the ink 1:1 with water and put the leaf into the solution for staining. After 3-4 minutes, take the leaf out. Rinse the leaf with clean water and dry the water.

After performing the above five steps, the leaf is ready for imaging purposes. A photo of the leaf is shown in Supplementary Fig. 2.

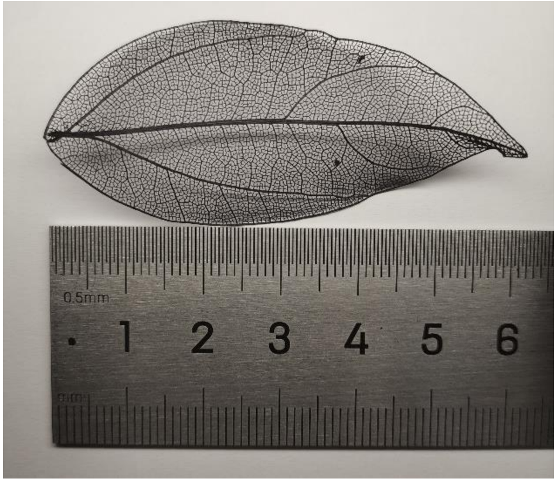

Supplementary Fig. 2. A photo of the leaf used for photoacoustic imaging.

Supplementary Note 3. Imaging reconstruction of the leaf vein with 1-sensor measurement

The reconstructed image in Fig. 3(b) of the main text was obtained using the 8th sensor in the micro-ring sensor array, which is the one at the center. Normally, different elements in the sensor array maintain different views of the sample. In this condition, a coherent summation of the information captured through these elements can improve the image quality by reducing artifacts due to a limited view. Nonetheless, since the center-to-center distance between different micro-ring sensors is only 400 μm and the sample was rotated during experiments, all micro-ring sensors, regardless of the ones at the edge or at the center, share roughly the same views of the sample. This

fact can be validated by examining the reconstructed images of the leaf vein achieved through different 1-sensor measurements. In particular, Supplementary Figs. 3(a), (b), (c), and (d) show the reconstructed images using only the 1st, the 4th, the 12th, and the 15th element, respectively. As shown in the figure, we could hardly see any considerable difference in the images reconstructed by different single micro-ring sensors. Compared with the one shown in the main text, the fluctuations in terms of the contrast-to-noise ratio of these images were within 7%, which we believe is most likely due to the variation in the sensitivity of different sensing elements.

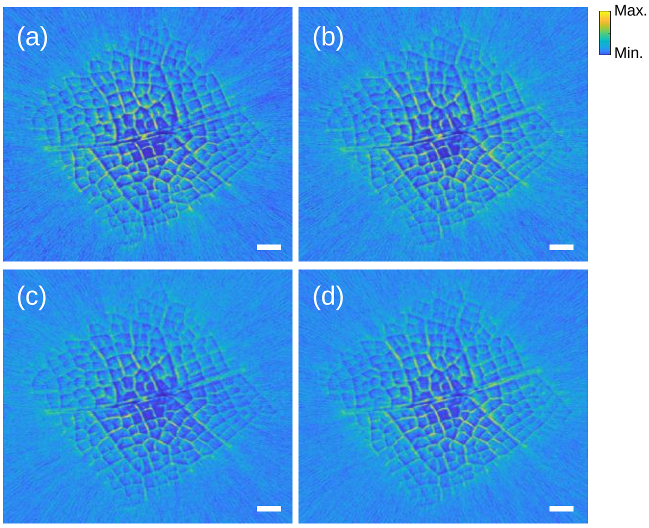

Supplementary Fig. 3. Reconstructed images of the leaf vein through different elements in the sensor array. Scale bars: 1 mm. (a) The 1st element; (b) the 4th element; (c) the 12th element; (d) the 15th element.

Supplementary Note 4. Comparison to other on-chip optical ultrasound sensors

In this section, we summarize some representative on-chip optical ultrasound sensors in Supplementary Tab. 1, which represent state-of-the-art performances. π-BG resonators fabricated through silicon photonics enable optical ultrasound sensors with a small footprint while maintaining very high sensitivity per unit area [2, 3]. On the other hand, the micro-ring can be conveniently extended with arrayed structures. To date, a silicon-based micro-ring sensor array with 10 elements [4] and a polymer-based micro-ring sensor array with 4 elements [5] were reported. However, parallel interrogation with one source-detector pair has not been realized. This work reports the fabrication of a chalcogenide-based micro-ring sensor array with 15 elements and demonstrates the first parallel interrogation for PAT with only one source-detector pair. Notably, the NEPs for all these optical ultrasound sensors are at roughly the same level. It is worth mentioning that the use of a digital optical frequency comb (DOFC) considerably increases the noise level, as discussed in Supplementary Note 11 in detail. Moreover, with parallel interrogation, there is also a compromise in the detecting bandwidth. For DOFC, the comb tooth spacing Δf is inversely proportional to the duration detecting time window T. Since T is the inverse of the sampling rate of the acoustic wave, it can be derived that the sampling rate is the same as the comb tooth spacing Δf. Thus, to meet the requirement of the Nyquist sampling theorem, the largest detecting bandwidth for acoustic waves with parallel interrogation is $\Delta f/2$. In our experiments, Δf = 39.0625 MHz, leading to a detecting bandwidth of about 20 MHz. Nonetheless, the tunability of the DOFC allows us to conveniently achieve a higher bandwidth by choosing a larger comb tooth spacing, which is at the cost of accuracy

in locating resonant frequencies.

Supplementary Tab. 1. Comparison of the state-of-the-art on-chip optical ultrasound sensors. H × W × L: height × weight × length; NEP: noise-equivalent pressure. Δf: comb tooth spacing.

	Material	Structure	Dimension (H×W×L, μm)	-6-dB Bandwidth (MHz)	NEP spectral density (mPa/Hz$^{-1/2}$)	NEP (Pa)	Sensor array (# of elements)	Parallel interrogation (PI)
Ref. [2]	Silicon	π-BG resonator	0.2×0.2×0.2	230	9	45	No	No
Ref. [3]	Silicon	π-BG resonator	0.2×0.5×30~200	200	2.2~9.8	/	No	No
Ref. [4]	Silicon	Micro-ring with membrane	0.2×15~20× 15~20	27	1.3~2	5.5	Yes (10)	No
Ref. [5]	Polymer	Micro-ring	1.4×60×60	350	5.61	105	Yes (4)	No
Ref. [6]			0.7×80×80	280	/	0.49	No	No
Ref. [7]			0.7×80×80	32	/	81	No	No
This work	Chalcogenide	Micro-ring	0.85×40×40	175 or Δf/2 (PI)	2.2 or 13.9 (PI)	7.1 or 36.9 (PI)	Yes (15)	Yes

Supplementary Note 5. Effects of claddings on chalcogenide-based micro-ring sensors

In this section, we describe the effects of claddings on the performance of chalcogenide-based micro-ring sensors. As a fair comparison, we chose three micro-ring sensors with roughly the same quality factors around 5×10^5, and encapsulated them with different claddings. As the control group, one micro-ring sensor has no cladding, which is referred to as the null case. The other two sensors were encapsulated with 3-µm-thick claddings using either polydimethylsiloxane (PDMS) or silicon dioxide (SiO_2). A schematic of the experimental setup to examine the effectiveness of the cladding is shown in Supplementary Fig. 4, in which acoustic waves directly impinge on the micro-ring sensors. It should be noted that compared to the transmitted power through micro-ring sensors with claddings, the final output power after transmitting through those micro-ring sensors without cladding decreases by a factor of about 2.3 dB due to water absorption. Thus, for a fair comparison, we adjusted the incident power such that the collected power at the receiver end is at the same level as 0.05 mW.

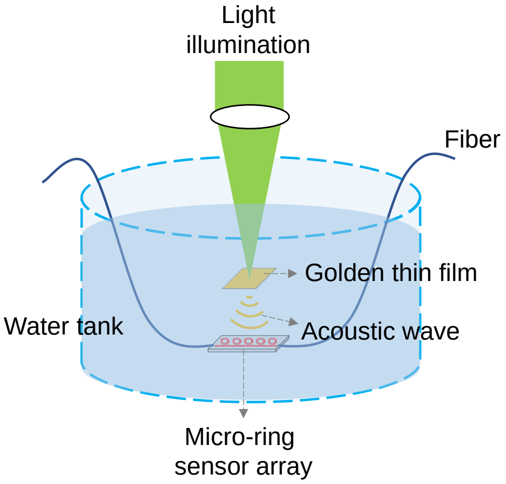

Supplementary Fig. 4. Schematics of the experimental setup to examine the effectiveness of the cladding.

Using the same characterization procedure described in the main text to produce Fig. 7(d), the amplitude maps of the measured signals as a function of time and translational distance are illustrated in Supplementary Fig. 5. As we can see from the figure, different claddings do not induce considerable differences in the amplitude maps and no strong surface acoustic wave is observed. Quantitatively, we found the peak values in these three cases are similar, indicating that signals are indeed contributed by the chalcogenide-based micro-ring structures. Therefore, we conclude that for micro-ring sensors made of soft chalcogenide-based material, the choices of cladding material do not considerably affect the performance of the sensor. It is worth noting that this observation is different from the one reported in Ref. [3], in which the sensor structure was made of a hard material SiO_2. In that case, the deformation in relatively soft PDMS cladding contributes to the measured signal.

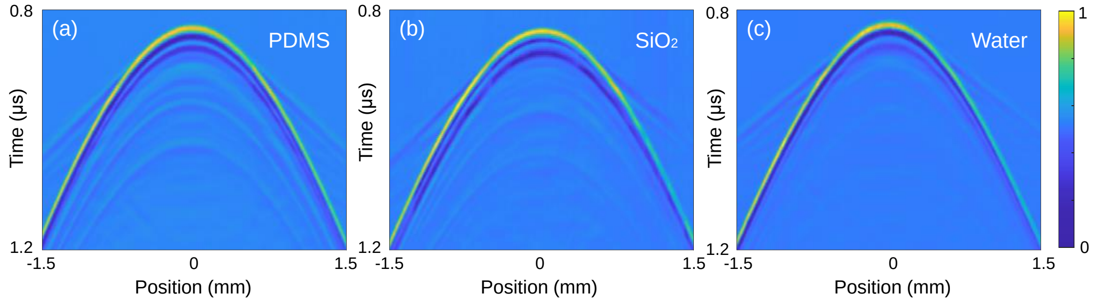

Supplementary Fig. 5. The amplitude maps of the measured signals as a function of time and translational distance. (a) With PDMS cladding. (b) With SiO_2 cladding. (c) Without cladding (water).

Supplementary Note 6. The procedures for fiber-to-chip bonding

In this section, we describe the procedures for fiber-to-chip bonding. The sensor chip was placed at a coupling platform, which was monitored by a microscope. The magnification of the eyepiece and objective lenses of the microscope were chosen to be 12× and 20×, respectively. Based on the shape and size of the propagating mode inside the bus waveguide, we chose to use a single-mode fiber with a mode fiber diameter of 3.2 ± 0.3 μm at 1550 nm (Nufern UHNA7, Coherent). Aided by the microscope, both ends of the bus waveguide were roughly aligned to the aforementioned type of single-mode fibers, as shown in Supplementary Fig. 6. This procedure was accomplished by adjusting the three-axis high-precision translation stage (MAX311D/M, Thorlabs) underneath the fiber. Note that a small gap was left between the waveguide and fiber for fine-tuning afterward.

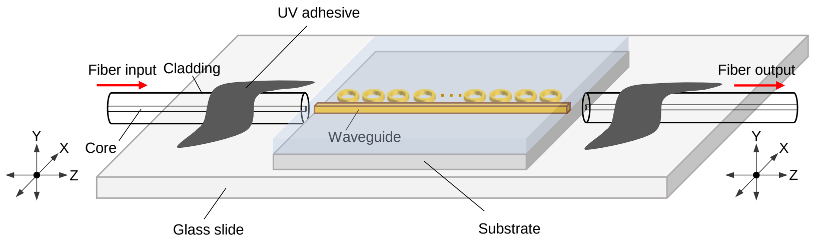

Supplementary Fig. 6. A schematic illustration of the fiber-to-chip bonding structure.

Then, the input side of the fiber was connected to a continuous-wave laser (Keysight, 8164B, 10-kHz linewidth, 1,550 nm), while the output side of the fiber was attached to an optical power meter (PM100D, Thorlabs) for fine-tuning. The two three-axis stages that support the two fibers were adjusted consecutively. When gradually decreasing the gap between the fiber and the waveguide along the z direction, the positions along the x and y directions were slightly adjusted to maximize the measurement of the power meter. As the fiber and the waveguide fit tightly, a small amount of ultraviolet curing adhesive (NOA61, Norland) with a refractive index of 1.56 was dripped at the connection point. An ultraviolet curing lamp (NVSUA U365nm, Nichia) with an illumination spot of 1.5 mm and a wavelength of 365 nm was used for solidifying the curing adhesive. Empirically, we chose an illumination time of 5 minutes and an illumination distance of 5 cm to guarantee satisfactory performance. Finally, the entire structure was moved and glued onto a glass slide. This additional glass slide further strengthens the stability of both the fibers and the chip. After this fine-tuning procedure, we experimentally quantified the insertion loss between the fiber and the waveguide was about 6 dB, leading to a total insertion loss of 12 dB for the sensor chip.

Supplementary Note 7. The generation and demodulation processes of the DOFC

In this section, we describe the details of the generation process of the DOFC, which is essential for realizing parallel interrogation to the micro-ring sensor array. Mathematically, the targeted comb structure in the frequency domain should read

$$D_{\text{input}}(f) = \sum_{q=-N/2}^{N/2} E_q \exp\left(j\phi_q\right) \cdot \delta\left(f - f_q\right) \quad \text{(S1)}$$

where E_q, ϕ_q, and f_q are the amplitude, phase, and frequency of the q-th comb line, respectively. $q = -N/2$, …, $N/2$ is used to denote the labeling of comb lines, where N is set as an even number. Moreover, we also have $f_q = f_0 + q\Delta f$, where f_0 is the carrier frequency of the light source and Δf is the comb spacing. It is worth noting that the comb tooths are evenly distributed on both sides of f_0. In practices, ϕ_q is set to 0 for all q, while E_q is set to a constant E_0. By taking the Fourier transformation, the corresponding waveform in the time domain is

$$E_{\text{DOFC}}(t) = \frac{1}{2\pi} \sum_{q=-N/2}^{N/2} E_0 \cos(2\pi(f_0 + q\Delta f)t) \quad \text{(S2)}$$

To generate the waveform in Eq. S2, one needs to modulate the optical field with the following form

$$E_{\text{M}}(t) = \frac{1}{\pi} \sum_{q=0}^{N/2} E_0 \cos(2\pi q\Delta f t) \quad \text{(S3)}$$

Supplementary Fig. 7 illustrates the experimental realization of the above mathematical descriptions. First, the computer generated a series of data as $D_{\text{input}}(f)$. Inverse fast Fourier transform (IFFT) was then conducted to generate $E_{\text{DOFC}}(t)$. After removing the carrier frequency f_0, a digital-to-analog converter (DAC) generated an electrical signal $E_{\text{M}}(t)$ and drove an intensity modulator (IM). Since the IM was set with a bias control at the null point (push-pull mode), both the positive and negative voltages represent large modulation depths. In this way, we effectively generated DOFC through the IM with the desired driving signal in Eq. (S3). As a result, the light output from a single-frequency laser was transformed into a frequency comb.

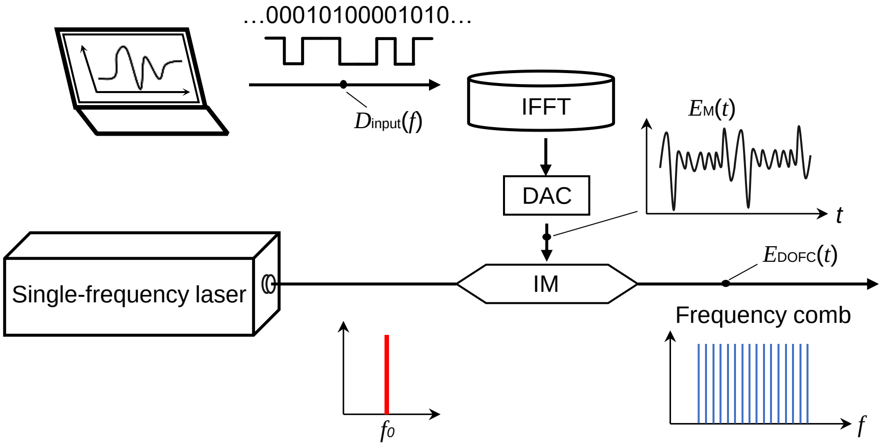

Supplementary Fig. 7. The generation process of the digital optical frequency comb. IFFT: inverse fast Fourier transformation; DAC: digital-to-analog converter; IM: intensity modulator.

Then, we proceed to describe the demodulation process of the DOFC. Having interacted with the micro-ring sensor array and acquired the information carried by ultrasonic wave, the optical field of the DOFC becomes

$$\tilde{E}_{\text{DOFC}}(t)=\frac{1}{2\pi}\sum_{q=-N/2}^{N/2}\tilde{E}_q\exp\big(j2\pi(f_0+q\Delta f)t+j\tilde{\phi}_q\big) \tag{S4}$$

Here, we use the notation tilde to denote the parameters of the DOFC after passing through the micro-ring sensor array. During experiments, coherent detection was employed to extract the following output optical field

$$\tilde{E}_{\text{output}}(t)=\frac{1}{2\pi}\sum_{q=-N/2}^{N/2}\tilde{E}_q\exp\big(j2\pi(q\Delta f)t+j\tilde{\phi}_q\big) \tag{S5}$$

where the optical frequency f_0 was filtered out. Then, we performed a fast Fourier transformation (FFT) to $\tilde{E}_{\text{output}}(t)$, leading to a data train as follows

$$\widetilde{D}_{\text{output}}(f)=\text{FFT}\big[\tilde{E}_{\text{output}}(t)\big]=\frac{1}{2\pi}\sum_{q=1}^{N}\tilde{E}_q\exp\big(j\tilde{\phi}_q\big)\cdot\text{FFT}[\exp(j2\pi(q\Delta f)t)]$$

$$=\sum_{q=-N/2}^{N/2}\tilde{E}_q\exp\big(j\tilde{\phi}_q\big)\cdot\delta\big(f-q\Delta f_q\big) \tag{S6}$$

Notably, the transmission spectrum of the micro-ring sensor array, which carries the information of the ultrasonic wave, can be obtained by dividing $\widetilde{D}_{\text{output}}(f)$ with respect to $D_{\text{input}}(f)$ as follows

$$T(f)=\big|\widetilde{D}_{\text{output}}(f)/D_{\text{input}}(f)\big|^2 \tag{S7}$$

Then, we swept the transmission spectrum to locate the positions of the resonant frequencies f_i where i is the labeling for micro-ring sensors. Taking the sensor array with 15 elements as an example, $i=1,2,3,\cdots,15$. In the absence of the ultrasonic wave, we recorded $f_i(0)$ as the original resonant frequency. When the ultrasonic wave interacts with the sensor array, all $f_i(t)$ start to change with time. In this condition, we define the time-dependent amplitude of the ultrasonic wave received by the i-th element $\text{PA}_i(t)$ as

$$\text{PA}_i(t)=f_i(t)-f_i(0) \tag{S8}$$

Thus, aided by the transmission spectrum measured through the DOFC, we can determine the time-dependent amplitude of the ultrasonic wave from all micro-ring sensors in parallel, without the need to lock single-wavelength lasers to the resonant frequencies as the conventional methods did.

Supplementary Note 8. The fabrication procedure for the golden layer

The fabrication procedure for the golden layer used for photoacoustic characterization is similar to that reported in Ref. [8]. The golden layer was fabricated under a high vacuum below 5×10^{-9} Torr using electron beam assisted deposition (DE400DUL, Detech). The substrate was chosen as Silicon dioxide with a thickness of 1,500 μm. Then, a 5-nm layer of titanium with a purity > 99.99% (Φ60 × 2 mm, ZhongNuo Advanced Material (Beijing) Technology Co., Ltd) was deposited at 0.3 Å /s on the substrate, serving as the adhesion layer. Subsequently, a 200-nm layer of gold with a purity > 99.99% (Φ60 × 2 mm, ZhongNuo Advanced Material (Beijing) Technology Co., Ltd) was deposited at the same speed. Such a relatively low deposition speed is to guarantee the high crystalline quality of the deposited metal layers. The entire procedure took about 2 hours.

Supplementary Note 9. Acceptance angles for the micro-ring sensor

Optical ultrasound sensors generally support wide acceptance angles. In this section, we detailed the characterization process for the acceptance angle of the micro-ring sensor. The experimental setup has been shown in Fig. 7(a) of the main text. In the beginning, the relative distance between the ultrasonic source and the micro-ring sensor was about 5 mm, and the time and

frequency responses at 0° are shown in Figs. 7(b) and (c) of the main text. Then, the micro-ring sensor was scanned in the horizontal direction. To generate a sufficient number of data to produce Fig. 7(d), the scanning step size was set to 5 μm. The translational distance can be converted into the acceptance angle through a simple trigonometric operation. The time responses at 0°, 10°, 20°, 30°, and 40° are provided in Supplementary Figs. 8(a)-(e). All signals were normalized to the maximum value of Supplementary Fig. 8(a). As we can see from the figure, a larger receiving angle leads to a smaller peak amplitude and a larger time delay. More importantly, signals get broadened for larger angles, indicating smaller central frequencies and narrower operating bandwidths. To illustrate this point, frequency responses at these corresponding angles are provided in Supplementary Figs. 8(f)-(j). Quantitatively, the 3-dB bandwidths are 115 MHz, 52 MHz, 21 MHz, 17 MHz, and 14 MHz at 0°, 10°, 20°, 30°, and 40°, respectively. Given that the 3-dB bandwidth at 0° is 115 MHz, we notice that the decreasing rate for the bandwidth of the sensor decays is fast for small angles and becomes slow for large angles.

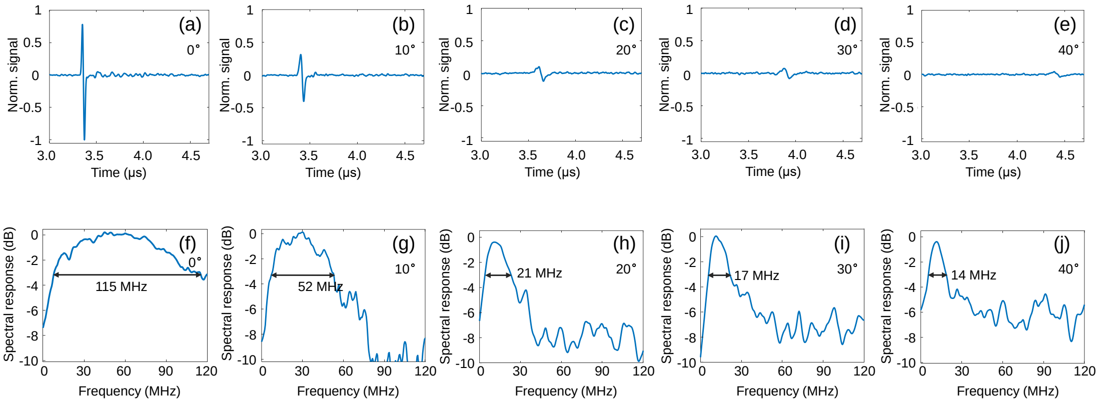

Supplementary Fig. 8. Characterization for the acceptance angle of the micro-ring sensor. (a)-(e) Time responses of the micro-ring sensor with an acceptance angle at 0°, 10°, 20°, 30°, and 40°, respectively. (f)-(j) Frequency responses of the micro-ring sensor with an acceptance angle at 0°, 10°, 20°, 30°, and 40°, respectively.

We also numerically investigated the theoretical angular response of the micro-ring sensor. In particular, we followed the procedure described in Ref. [9] to examine the spatial distribution of ultrasonic detection. To comply with the parameters used in experiments, the diameter of the micro-ring sensor was set to 40 μm and the relative distance between the ultrasonic point source and the sensor at the beginning was 5 mm during simulations. The frequency response of the micro-ring sensor as a function of the acceptance angle is shown in Supplementary Fig. 9. Two red -3 dB lines are also provided for visualization purposes. This figure confirms the experimental results presented in Fig. 7(e) show similar trends to the theoretical ones.

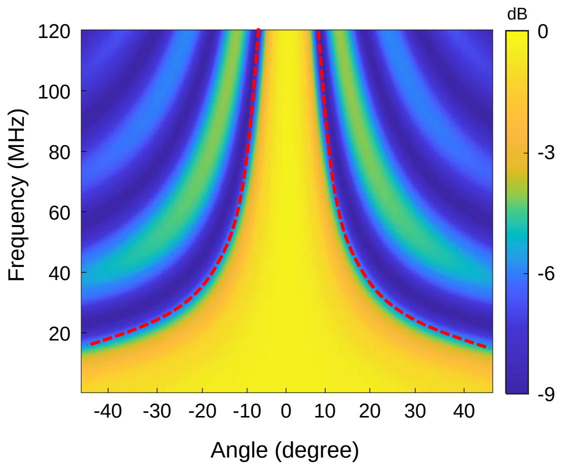

Supplementary Fig. 9. Numerical investigation of the theoretical angular response of the micro-ring sensor.

Supplementary Note 10. Characterization process of the sensitivity of the micro-ring sensor

In this section, we describe the characterization process of the sensitivity of the micro-ring sensor, which follows the conventional procedure described in Ref. [4]. The ultrasound was generated by a piezo-electric transducer (Olympus, V312) with a central frequency of 10 MHz. The transducer was driven by a rectangular signal with a duration of 40 ns and a peak voltage of 5 V. A single photodetector (Agilent, 11982A) was employed to collect the transmitted optical signal. We first placed a calibrated needle hydrophone (Precision Acoustics, NH0200, 20-MHz bandwidth) 2.5-mm away from the source to measure the ultrasonic wave. The measured acoustic signal $H(t)$ as a function of time is shown in Supplementary Fig. 10(a), which was averaged through 200 independent time traces. Its Fourier transformation $H(f)$, which is known as the amplitude spectral density, is plotted in Supplementary Fig. 10(b). Thus, the generated pressure spectral density $P(f)$ can be estimated as (Supplementary Fig. 10(c))

$$P(f) = H(f)/S_{\mathrm{H}}(f) \tag{S9}$$

where $S_{\mathrm{H}}(f)$ is the sensitivity of the calibrated Hydrophone (provided by the company). To characterize the sensitivity of the micro-ring sensor, we placed it at the same position as the calibrated hydrophone and triggered the same ultrasonic wave. Similarly, the measured acoustic signal $M(t)$ as a function of time is shown in Supplementary Fig. 10(d). By performing Fourier transformation, the amplitude spectral density of the micro-ring sensor $M(f)$ is shown in Supplementary Fig. 10(e). Therefore, the sensitivity of the micro-ring sensor $S_{\mathrm{M}}(f)$ can be estimated as

$$S_{\mathrm{M}}(f) = M(f)/P(f) \tag{S10}$$

which is shown in Supplementary Fig. 10(f).

Having obtained the sensitivity of the micro-ring sensor, we further characterized the noise-equivalent pressure (NEP) spectral density $N_{\mathrm{Pa}}(f)$ (Fig. 7(g) in the main text). This quantity can be estimated as

$$N_{\mathrm{Pa}}(f) = N_{\mathrm{V}}(f)/S_{\mathrm{M}}(f) \tag{S11}$$

Here, $|N_{\mathrm{V}}(f)|^2$ is the noise power spectral density, which was computed through a 10-μs long-time trace without ultrasonic excitation (Fig. 7(f) in the main text). Specifically, it was estimated using the Welch method with segments of 1,400 samples, the Hanning windows, and 512 samples overlapping [10]. Moreover, the root-mean-square (RMS) pressure $P_{\mathrm{NEP,RMS}}$ within the range from

0 to 20 MHz was computed as

$$P_{\text{NEP,RMS}} = \sqrt{\int_0^{20\ \text{MHz}} |N_{\text{Pa}}(f)|^2 df} \tag{S12}$$

which is about 7.1 Pa.

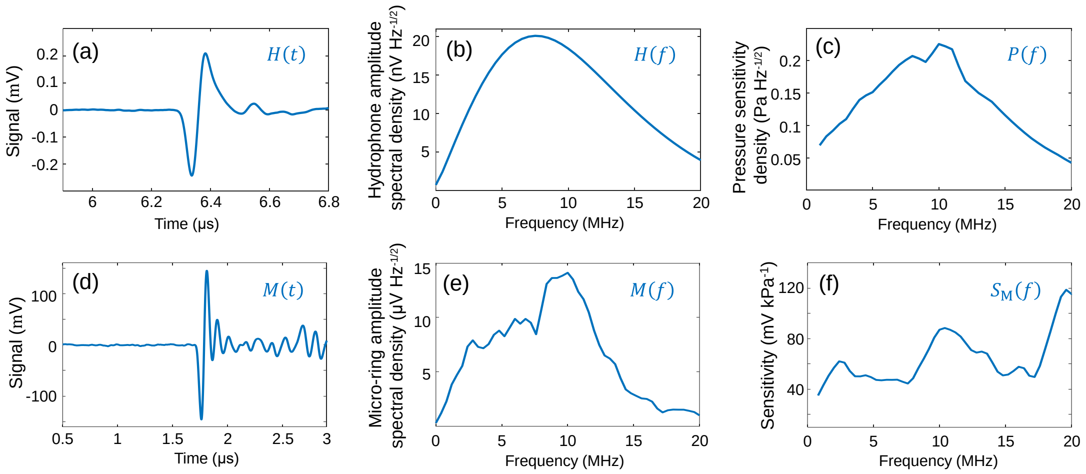

Supplementary Fig. 10. Characterization process of the sensitivity of the micro-ring sensor. (a) The measured acoustic signal $H(t)$ as a function of time using a calibrated hydrophone. (b) The amplitude spectral density $H(f)$ measured by the calibrated hydrophone. (c) The generated pressure spectral density $P(f)$ by the piezo-electric ultrasonic transducer. (d) The measured acoustic signal $M(t)$ as a function of time using the micro-ring sensor. (e) The amplitude spectral density $M(f)$ measured by the micro-ring sensor. (f) The sensitivity of the micro-ring sensor $S_{\text{M}}(f)$.

Supplementary Note 11. Noise-equivalent pressure with the parallel interrogation method

The parallel interrogation method certainly introduces additional noise, thus increasing the NEP of the measurement. In this section, we detail the quantification process of the NEP with the parallel interrogation method. Following the similar procedure described in Supplementary Note 10, the amplitude spectral density of the micro-ring sensor $M^{\text{PI}}(f)$ is shown in Supplementary Fig. 11(a). It is worth mentioning that the parallel interrogation method adopts the spectral shift (in the unit of MHz) as the indicator for the strength of ultrasound. By using the same calibrated needle hydrophone, the sensitivity of the micro-ring sensor $S_{\text{M}}^{\text{PI}}(f)$ was estimated and shown in Supplementary Fig. 11(b). Similarly, the noise amplitude spectral density $N_{\text{V}}^{\text{PI}}(f)$ was also quantified using the spectral shift, which is shown in Supplementary Fig. 11(c). With these parameters, the NEP spectral density $N_{\text{Pa}}^{\text{PI}}(f)$ can be estimated as

$$N_{\text{Pa}}^{\text{PI}}(f) = N_{\text{V}}^{\text{PI}}(f)/S_{\text{M}}^{\text{PI}}(f) \tag{S13}$$

This parameter is shown in Supplementary Fig. 11(d), which is always below 13.9 mPaHz$^{-1/2}$. The RMS pressure within the range from 0 to 20 MHz was computed be to 36.9 Pa, which is larger than the NEP of a single micro-ring quantified above using the conventional approach.

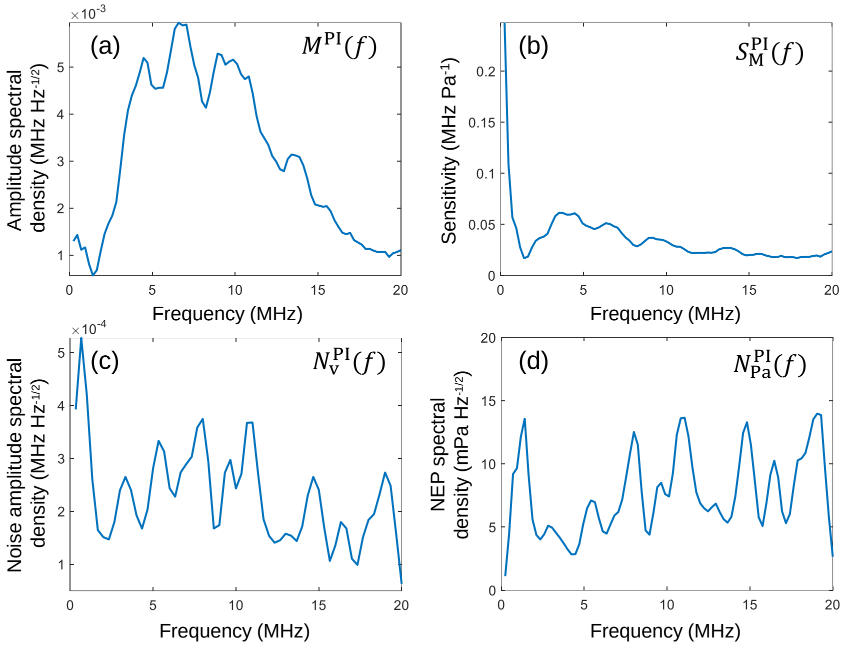

Supplementary Fig. 11. Quantification of the noise-equivalent pressure (NEP) of the micro-ring sensor with parallel interrogation. (a) The amplitude spectral density $M^{\mathrm{PI}}(f)$. (b) The sensitivity of the micro-ring sensor $S_{\mathrm{M}}(f)$. (c) The noise amplitude spectral density $N_{\mathrm{V}}^{\mathrm{PI}}(f)$. (d) The NEP spectral density $N_{\mathrm{Pa}}^{\mathrm{PI}}(f)$.

Supplementary Note 12 Numerical simulations on the point spread function (PSF) of the imaging system

In this section, we employed a numerical tool (k-wave, Version 1.2.1) to simulate the PSF of the imaging system, which manifests the artifacts due to limited view and reduced bandwidth as a function of angles. The simulation is in a two-dimensional plane and the geometrical parameters adopted in this simulation are identical to the experimental conditions presented in the main text. Further, we used the data presented in Fig. 7(e) of the main text to confine the angular response during simulations. Simulation results along both lateral and axial directions are shown in Supplementary Figs. 12(a) and (b), exhibiting similar trends as the ones obtained experimentally in Figs. 7(i) and (j), respectively. Moreover, the determined lateral and axial resolutions from experiments and simulations are found to be quantitively close.

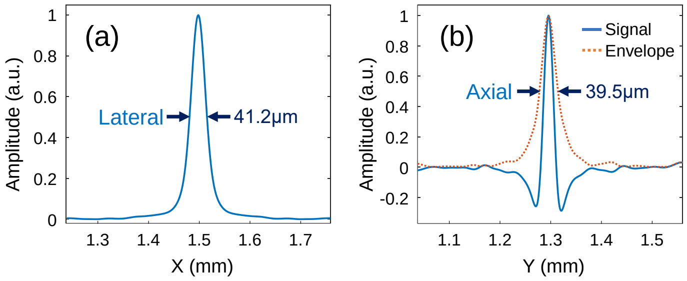

Supplementary Fig. 12. Numerical simulations on the PSF of the imaging system. (a) One-dimensional profile along the lateral direction, suggesting a lateral resolution of 41.2 μm. (b) One-dimensional profile along the axial direction, suggesting an axial resolution of 39.5 μm.

Supplementary Note 13. The process of frequency tuning for the micro-ring sensor array

The chalcogenide glass is an important class of semiconductor compounds that generally contains one or several types of chalcogenide elements, such as S, Se, or Te. In these compounds, chalcogenide elements form network structures with elements like Ge, Sb, or As through covalent bonds. Chalcogenide glasses usually exhibit a strong photosensitive effect, which is generally induced by structural rearrangement due to photon absorption [11]. Structural rearrangements, such as bending, breaking, and reforming, have been observed in many types of chalcogenide glasses, leading to a refractive index change in the macroscopic picture [12].

To exploit the photosensitive effect for frequency tuning, we built an experimental shown in Supplementary Fig. 13(a), which consists of a pump laser, an optical fiber, a three-dimensional motorized translational stage, a microscope system, and a tray to hold the micro-ring sensor array. We first quantified the photosensitivity of the chalcogenide micro-ring sensor through 532-nm light illumination at 30 mW. Supplementary Fig. 13(b) plots the observed resonant wavelength shift as a function of illumination time, exhibiting an exponential behavior. Moreover, after being illuminated for 120 s, the resonant wavelength can be shifted by an amount of up to 7.25 nm. This value is large enough to cover almost the entire free spectral range of the micro-ring resonator λ_{FSR} = 8.5 nm, which can be estimated through $\lambda_0^2/2\pi nR$. Here λ_0 = 1,550 nm is the operating wavelength, n = 2.33 is the refractive index of the chalcogenide glass, and $R = 20$ μm is the radius of the micro-ring sensor.

With these preliminary results, we then proceed to tune the resonant frequencies of these micro-ring sensors made of chalcogenide glass. A typical illustrative transmission spectrum of the sensor array is depicted in Supplementary Fig. 13(c). In general, their resonant frequencies are not positioned in order and may even cover a wide frequency range beyond 40 GHz (the bandwidth of the DOFC). This condition, as mentioned in the main text, is unfavorable for the DOFC-enabled parallel interrogation. To address this issue, we need to order and equally space these resonant frequencies by utilizing the photosensitive effect and the experimental setup in Fig. 12(b). The tunning process for resonant frequencies is described as follows. 1. We control the size of the illumination light so that it matches the diameter of the micro-ring sensor. 2. Under the illumination of low-intensity light, we adjust the height and position of the optical fiber so that light illumination acts on only one micro-ring sensor. This process is performed with the assistance of the microscope system. 3. While monitoring its resonant condition through a frequency-swept detection system (not shown in Figure), we increase the output power of the illuminating light to tune the resonant frequency of this micro-ring sensor. In order to efficiently utilize the bandwidth of the DOFC, the resonant frequency of the first micro-ring sensor is tuned to the far end of the operating window. 4. After tuning the resonant frequency of the targeted micro-ring sensor to the desired value, the illuminating light is turned off and the three-dimensional motorized precision translational stage transports the next micro-ring sensor to the illumination area for frequency tuning. 5. The tuning process is repeated for each micro-ring sensor in order until all resonant frequencies are placed to their designed values. An illustrative transmission spectrum of the micro-ring sensor array after the tuning process is shown in Supplementary Fig. 13 (d), exhibiting ordered and equally spaced resonant frequencies.

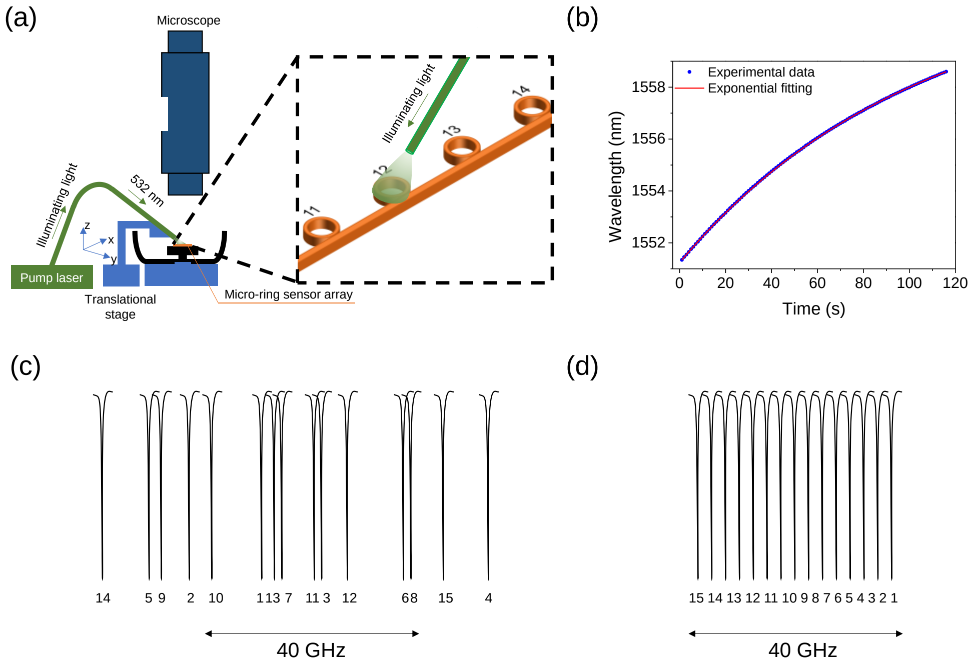

Supplementary Fig. 13. Tuning the resonant frequencies for the chalcogenide-based micro-ring sensor array through the photosensitive effect. (a) Experimental setup of the tuning process. (b) The measured resonant wavelength shift as a function of illumination time for a typical micro-ring sensor. (c) An illustrative transmission spectrum of the sensor array before frequency tuning. (d) An illustrative transmission spectrum of the sensor array after frequency tuning.

Supplementary Note 14. The examination of the spectrum stability of the micro-ring sensor array

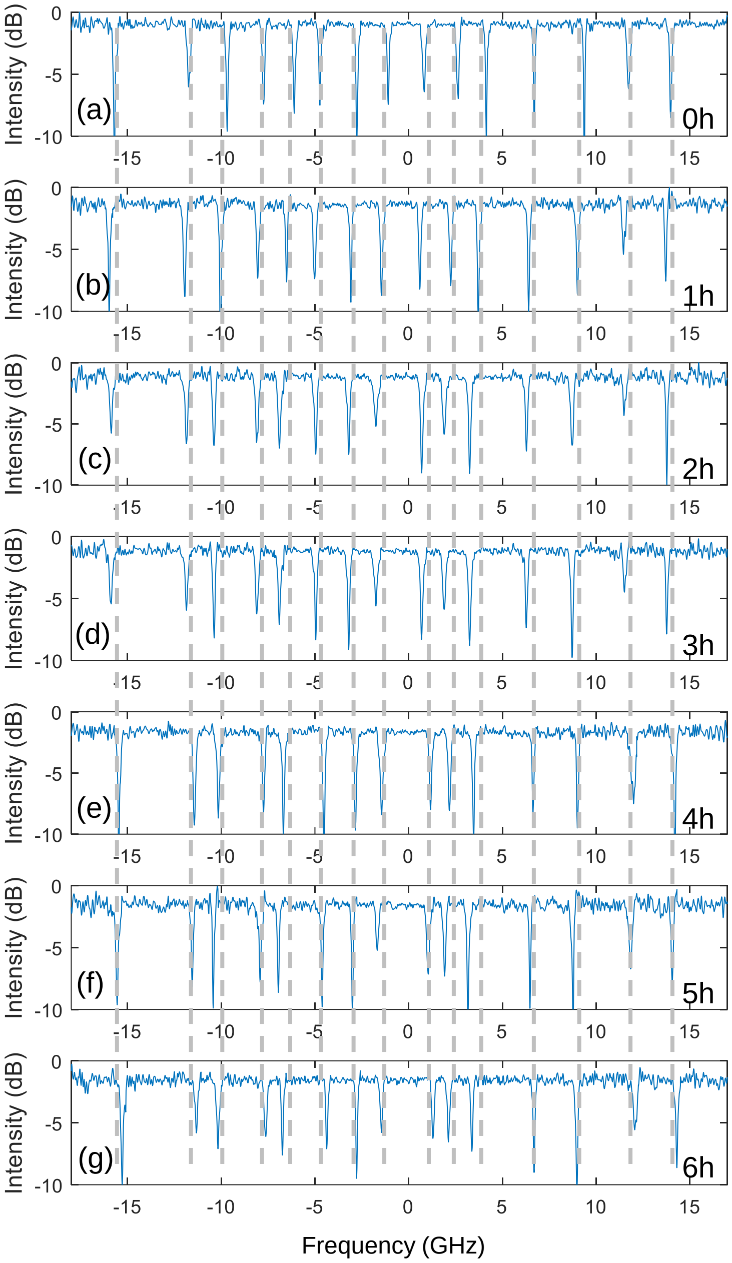

Supplementary Fig. 14. The measured transmission spectra for the micro-ring sensor array over time. The original transmission spectrum (a). Transmission spectra measured after 1 hour (b), 2 hours (c), 3 hours (d), 4 hours (e), 5 hours (f), and 6 hours (g).

The DOFC method strongly relies on the delicate tuning of the sensor spectrum. In this section, we examined the spectrum stability of the micro-ring sensor array. Thanks to the employment of the DOFC, each comb tooth effectively acts as an equally spaced sampling point, serving as a tool to quantify the entire transmission spectrum of the sensor array even when the shifted resonant frequencies become disordered. Firstly, we measured the transmission spectrum of the sensor array over time for 6 hours, which is shown in Supplementary Fig. 14. As we can see from the figure, the photo-sensitive effect of the material did cause observable resonant frequency shifts in the spectrum. Nonetheless, all 15 resonant dips are still well-resolvable, allowing sustainable imaging operation using the sensor array. For comparison purposes, grey dashed lines were used to denote the position

of the original resonant frequencies. Quantitatively, the mean absolute frequency drifts of the 15 resonant frequencies after 1 hour, 2 hours, 3 hours, 4 hours, 5 hours, and 6 hours are 0.319 GHz, 0.451 GHz, 0.451 GHz, 0.312 GHz, 0.401 GHz, and 0.375 GHz, respectively. Correspondingly, the standard deviation of the frequency drifts of the 15 resonant frequencies after 1 hour, 2 hours, 3 hours, 4 hours, 5 hours, and 6 hours are 0.067 GHz, 0.267 GHz, 0.267 GHz, 0.188 GHz, 0.309 GHz, and 0.208 GHz, respectively. All these values are much smaller than the average separation of adjacent resonant dips (1.66 GHz). These results show that the micro-ring sensor array can still function normally even after being placed in the aqueous environment for 6 hours. However, for experiments on different days, we still need to tune the resonance spectrum of the sensor array before each experiment. For future applications that require long stability, we anticipate that the photosensitive effect can be possibly mitigated by adjusting the chemical composition of the chalcogenide glass, which is out of the scope of this work.

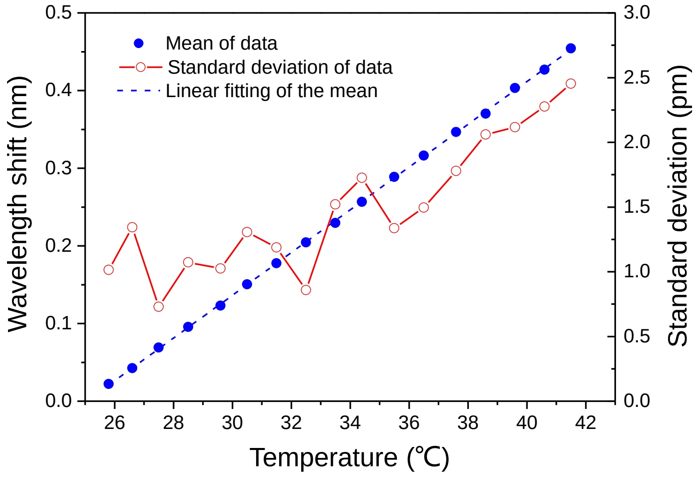

Supplementary Fig. 15. The measured resonant wavelength shift for micro-ring sensors as a function of temperature. The blue dots and red circles represent the mean values and standard deviations from the measurements of these 15 micro-rings. The blue dashed line denotes the linear fitting of the mean.

In addition, the temperature change of water could also resonant frequency shifts. To examine this issue, we varied the temperature of the aqueous environment within a range from 25 - 40 °C by using a heating pad. Notably, it was observed that all 15 sensors in the array exhibited roughly the same frequency drifts along the same direction. This observation indicates that the variation in the temperature of the water does not scramble the spectrum of the sensor array. The mean resonant frequency shifts for these 15 resonant frequencies are plotted in Supplementary Fig. 15 as a function of temperature using blue dots, while their standard deviations are represented using red circles. As we can see from the figure, the frequency drifts increase linearly with the increased temperature. Through a linear fitting, the measured data results in a thermo-optic coefficient of about 25.9 pm/°C (blue dashed line), which is in agreement with the one reported in the literature. Notably, these wavelength shifts are already larger than the full width at half maximum (FWHM) of typical resonant dips. Such wavelength shifts may cause difficulties for typical conventional methods that generally require the wavelength shift to be smaller than the FWHM of the resonant dip [13]. Nonetheless, the relatively large wavelength shift may not become a big problem with the employment of the DOFC, as it provides a complete transmission spectrum within a 40-GHz range. As long as the resonant frequency does not go beyond this range, we can always precisely locate

the position of the resonant frequency. The only criterion applies for parallel interrogation of these micro-ring sensor arrays, which requires adjacent resonant frequencies to be identifiable. In other words, the frequency separation between any two adjacent resonant frequencies should be larger than the FWHM of the resonant dip. No further restriction on the absolute wavelength shifts is enforced.

Reference

1. M. Xu and L. V. Wang, "Universal back-projection algorithm for photoacoustic computed tomography," Physical Review E **71**, 016706 (2005).
2. R. Shnaiderman, G. Wissmeyer, O. Ülgen, Q. Mustafa, A. Chmyrov, and V. Ntziachristos, "A submicrometre silicon-on-insulator resonator for ultrasound detection," Nature **585**, 372-378 (2020).
3. Y. Hazan, A. Levi, M. Nagli, and A. Rosenthal, "Silicon-photonics acoustic detector for optoacoustic micro-tomography," Nature Communications **13**, 1488 (2022).
4. W. J. Westerveld, M. Mahmud-Ul-Hasan, R. Shnaiderman, V. Ntziachristos, X. Rottenberg, S. Severi, and V. Rochus, "Sensitive, small, broadband and scalable optomechanical ultrasound sensor in silicon photonics," Nature Photonics **15**, 341-345 (2021).
5. C. Zhang, S. L. Chen, T. Ling, and L. J. Guo, "Review of Imprinted Polymer Microrings as Ultrasound Detectors: Design, Fabrication, and Characterization," IEEE Sensors Journal **15**, 3241-3248 (2015).
6. H. Li, B. Dong, X. Zhang, X. Shu, X. Chen, R. Hai, D. A. Czaplewski, H. F. Zhang, and C. Sun, "Disposable ultrasound-sensing chronic cranial window by soft nanoimprinting lithography," Nature Communications **10**, 4277 (2019).
7. Q. Rong, Y. Lee, Y. Tang, T. Vu, C. Taboada, W. Zheng, J. Xia, D. A. Czaplewski, H. F. Zhang, C. Sun, and J. Yao, "High-Frequency 3D Photoacoustic Computed Tomography Using an Optical Microring Resonator," BME Frontiers **2022**(2022).
8. M. Seeger, D. Soliman, J. Aguirre, G. Diot, J. Wierzbowski, and V. Ntziachristos, "Pushing the boundaries of optoacoustic microscopy by total impulse response characterization," Nature Communications **11**, 2910 (2020).
9. B. Dong, C. Sun, and H. F. Zhang, "Optical Detection of Ultrasound in Photoacoustic Imaging," IEEE Transactions on Biomedical Engineering **64**, 4-15 (2017).
10. P. Welch, "The use of fast Fourier transform for the estimation of power spectra: a method based on time averaging over short, modified periodograms," IEEE Transactions on audio and electroacoustics **15**, 70-73 (1967).
11. A. Zakery and S. R. Elliott, "Optical properties and applications of chalcogenide glasses: a review," Journal of Non-Crystalline Solids **330**, 1-12 (2003).
12. N. Singh, D. D. Hudson, R. Wang, E. C. Mägi, D.-Y. Choi, C. Grillet, B. Luther-Davies, S. Madden, and B. J. Eggleton, "Positive and negative phototunability of chalcogenide (AMTIR-1) microdisk resonator," Opt. Express **23**, 8681-8686 (2015).
13. C. Zhang, T. Ling, S.-L. Chen, and L. J. Guo, "Ultrabroad bandwidth and highly sensitive optical ultrasonic detector for photoacoustic imaging," Acs Photonics **1**, 1093-1098 (2014).
