# Supplementary material for: Parallel interrogation of the chalcogenide-based micro-ring sensor array for photoacoustic tomography
Source: Nat Commun. 2023 Jun 5;14:3250. doi: 10.1038/s41467-023-39075-3 (PMC10241812; doi:10.1038/s41467-023-39075-3)
Supplement: Supplementary file 2 — Reporting Summary [file 41467_2023_39075_MOESM2_ESM.pdf]

nature portfolio

Corresponding author(s): Yuecheng Shen and Zhaohui Li

Last updated by author(s): May 16, 2023

Reporting Summary

Nature Portfolio wishes to improve the reproducibility of the work that we publish. This form provides structure for consistency and transparency in reporting. For further information on Nature Portfolio policies, see our Editorial Policies and the Editorial Policy Checklist.

Statistics

For all statistical analyses, confirm that the following items are present in the figure legend, table legend, main text, or Methods section.

n/a	Confirmed	
☐	☒	The exact sample size (n) for each experimental group/condition, given as a discrete number and unit of measurement
☒	☐	A statement on whether measurements were taken from distinct samples or whether the same sample was measured repeatedly
☒	☐	The statistical test(s) used AND whether they are one- or two-sided<br>*Only common tests should be described solely by name; describe more complex techniques in the Methods section.*
☒	☐	A description of all covariates tested
☒	☐	A description of any assumptions or corrections, such as tests of normality and adjustment for multiple comparisons
☐	☒	A full description of the statistical parameters including central tendency (e.g. means) or other basic estimates (e.g. regression coefficient) AND variation (e.g. standard deviation) or associated estimates of uncertainty (e.g. confidence intervals)
☒	☐	For null hypothesis testing, the test statistic (e.g. F, t, r) with confidence intervals, effect sizes, degrees of freedom and P value noted<br>*Give P values as exact values whenever suitable.*
☒	☐	For Bayesian analysis, information on the choice of priors and Markov chain Monte Carlo settings
☒	☐	For hierarchical and complex designs, identification of the appropriate level for tests and full reporting of outcomes
☒	☐	Estimates of effect sizes (e.g. Cohen's d, Pearson's r), indicating how they were calculated

Our web collection on statistics for biologists contains articles on many of the points above.

Software and code

Policy information about availability of computer code

Data collection	Data was measured by a coherent receiver (Finisar, CPRV122xA) and digitized by an oscilloscope (Lecroy, 10-36Zi-A).
Data analysis	A digital signal processing unit performed a Fourier transformation to the measured signal and reconstructed the transmission spectrum of the sensor array. Photoacoustic signals were retrieved through the reconstructed transmission spectrum. All required codes are provided in "code_files.zip" at https://doi.org/10.5281/zenodo.7481865.

For manuscripts utilizing custom algorithms or software that are central to the research but not yet described in published literature, software must be made available to editors and reviewers. We strongly encourage code deposition in a community repository (e.g. GitHub). See the Nature Portfolio guidelines for submitting code & software for further information.

Data

Policy information about availability of data

All manuscripts must include a data availability statement. This statement should provide the following information, where applicable:

- Accession codes, unique identifiers, or web links for publicly available datasets
- A description of any restrictions on data availability
- For clinical datasets or third party data, please ensure that the statement adheres to our policy

The imaging data generated in this study have been deposited in the Zenodo database under accession code https://doi.org/10.5281/zenodo.7688935.

Human research participants

Policy information about studies involving human research participants and Sex and Gender in Research.

Reporting on sex and gender	Not applicable
Population characteristics	Not applicable
Recruitment	Not applicable
Ethics oversight	Not applicable

Note that full information on the approval of the study protocol must also be provided in the manuscript.

Field-specific reporting

Please select the one below that is the best fit for your research. If you are not sure, read the appropriate sections before making your selection.

☒ Life sciences ☐ Behavioural & social sciences ☐ Ecological, evolutionary & environmental sciences

For a reference copy of the document with all sections, see nature.com/documents/nr-reporting-summary-flat.pdf

Life sciences study design

All studies must disclose on these points even when the disclosure is negative.

Sample size	The main goal of this work is to demonstrate the imaging capability of the micro-ring sensor array by targeting different biological samples. In this study, we chose to present the imaging data for one piece of leaf and three zebrafish at different growth stages (7 days post-fertilization, 20 days post-fertilization, and 3-month-old). No specific sample size calculation was performed, as we believe different types of biological samples with various properties and sizes can validate the imaging performance of the micro-ring sensor array.
Data exclusions	No data was excluded from the analyses.
Replication	Every time we calibrate the system, the photoacoustic images of these biological tissues can be directly replicated. For each sample, the replication can be performed independently. Example data and reconstruction codes were provided alongside to allow readers to replicate our results.
Randomization	During the sample preparation process, at least three pieces of leaves were prepared. Moreover, for zebrafish at different growth stages, we prepared at least 10 for each group. Biological samples belonging to the same group have no big difference visually. During experiments, we randomly pick one of them from each group for demonstrating the imaging capability of the micro-ring sensor array. We declare that there is no predetermination of samples during our experiments.
Blinding	The investigators were blinded to group allocation during data collection and analysis.

Reporting for specific materials, systems and methods

We require information from authors about some types of materials, experimental systems and methods used in many studies. Here, indicate whether each material, system or method listed is relevant to your study. If you are not sure if a list item applies to your research, read the appropriate section before selecting a response.

Materials & experimental systems

n/a	Involved in the study	
☒	☐	Antibodies
☒	☐	Eukaryotic cell lines
☒	☐	Palaeontology and archaeology
☐	☒	Animals and other organisms
☒	☐	Clinical data
☒	☐	Dual use research of concern

Methods

n/a	Involved in the study	
☒	☐	ChIP-seq
☒	☐	Flow cytometry
☒	☐	MRI-based neuroimaging

Animals and other research organisms

Policy information about studies involving animals; ARRIVE guidelines recommended for reporting animal research, and Sex and Gender in Research

Laboratory animals	biological samples (zebrafish) used for photoacoustic imaging in this study are at the different growth stages: 3-month-old, 7 days post-fertilization, and 20 days post-fertilization.
Wild animals	The study did not involve wild animals.
Reporting on sex	The finding is not relevant to sex.
Field-collected samples	The study did not involve samples collected from the field.
Ethics oversight	Institutional Animal Care Committee of Sun Yat-sen University.

Note that full information on the approval of the study protocol must also be provided in the manuscript.
